# Unusual Case of Papillary Carcinoma of the Breast

**DOI:** 10.7759/cureus.63568

**Published:** 2024-07-01

**Authors:** Anuradha Dnyanmote, Himashree M.P.

**Affiliations:** 1 Department of General Surgery, Dr. D. Y. Patil Medical College, Hospital and Research Centre, Pune, IND

**Keywords:** rare breast tumors, fibrovascular cores, aggresive tumor breast, papaillary carcinoma breast, invasive breast carcinoma

## Abstract

Papillary carcinoma of the breast represents a distinct subtype of breast cancer characterized by its unique clinical and histopathological features. It is seen predominantly affecting post-menopausal women. Overall, papillary carcinoma has a low prevalence.

Histologically, papillary carcinoma is characterized by the presence of papillae-like structures lined by epithelial cells and supported by fibrovascular cores, appearing like a dual-layered epithelium. The clinical, epidemiological, and pathological characteristics of papillary carcinoma of the breast are not widely described in the existing literature.

The gold standard for diagnosis of carcinoma breast of any type remains a core needle biopsy, but in our case, the diagnosis of papillary carcinoma was made in the final histopathology specimen. Treatment strategies for papillary carcinoma include surgical excision with or without axillary dissection followed by adjuvant chemo-radiotherapy depending on the immunohistochemistry and tumor characteristics, but there appear multiple variations in the management of more common NOS (not otherwise specified) type and the papillary carcinoma of the breast. We present a case report of this papillary carcinoma of the breast.

## Introduction

Among aggressive breast carcinomas, less than 0.5% are papillary breast tumors, which usually manifest as an abnormal lump, bloody nipple discharge, or abnormalities on radiographs [1,2]. The histological demonstration shows a solid mass formed by cell proliferation that is grouped around a fibrovascular core.

It is important to differentiate between invasive and non-invasive forms of papillary cancer since each has a distinct prognosis. However, because of their low cumulative frequency, large series frequently include a combination of these two diagnoses [3]. Furthermore, difficulty in characterizing papillary carcinoma stems from the fact that individual case reports constitute the majority of the literature on this entity. We present a rare case of papillary carcinoma of the left breast in a post-menopausal woman.

## Case presentation

A 65-year-old female with no co-morbidities presented with a lump in the left breast for one month. The lump was insidious in onset, progressive, and not associated with a history of trauma or pain. There was no history of purulent/bloody nipple discharge from the affected side, and there were no lumps noted in the opposite breast by history. There was no associated history of fever or lumps elsewhere in the body.

**Figure 1:**
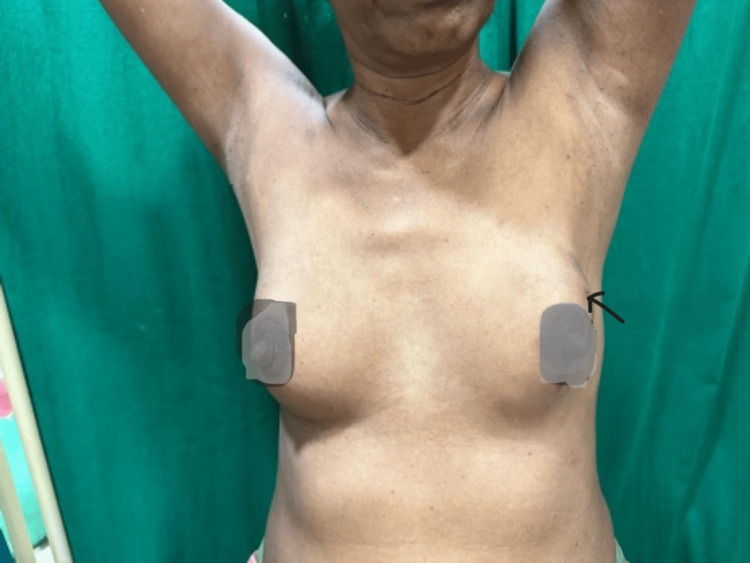
Clinical photo suggesting fullness seen on the left breast

On an examination of the breast, the skin over the upper outer quadrant of the left breast appeared tethered and the nipple was raised at a higher level compared to the right breast. There was evidence of puckering present in the skin over the left breast. Puckering occurs due to the involvement of Cooper's ligament and is more in favor of malignancy. On palpation, there was a lump measuring 4 x 5 cm in the upper outer quadrant with variable consistency, fixed within the breast tissue but not to the underlying chest wall; movement restriction was noted on putting the pectoralis muscle into contraction. There were no palpable lymph nodes noted in the bilateral axilla or cervical region. Figure 1 depicts the clinical photo of the patient.

A clinical diagnosis of breast carcinoma was established, prompting further investigation. Full-field digital mammography demonstrated an irregular, hyperdense mass with a cystic component located in the upper outer quadrant, measuring 44 x 31 mm. The mass exhibited irregular margins and fine pleomorphic calcifications in certain areas, indicative of malignancy. An ultrasound-guided fine-needle aspiration cytology (FNAC) was performed, revealing atypical cells, and the lesion was assigned a BI-RADS (Breast Imaging Reporting and Data System) score of 4C. Consequently, a Tru-Cut biopsy was conducted, which also revealed atypical cells with increased pleomorphism. However, no definitive evidence of invasion to confirm malignancy was observed.

**Figure 2:**
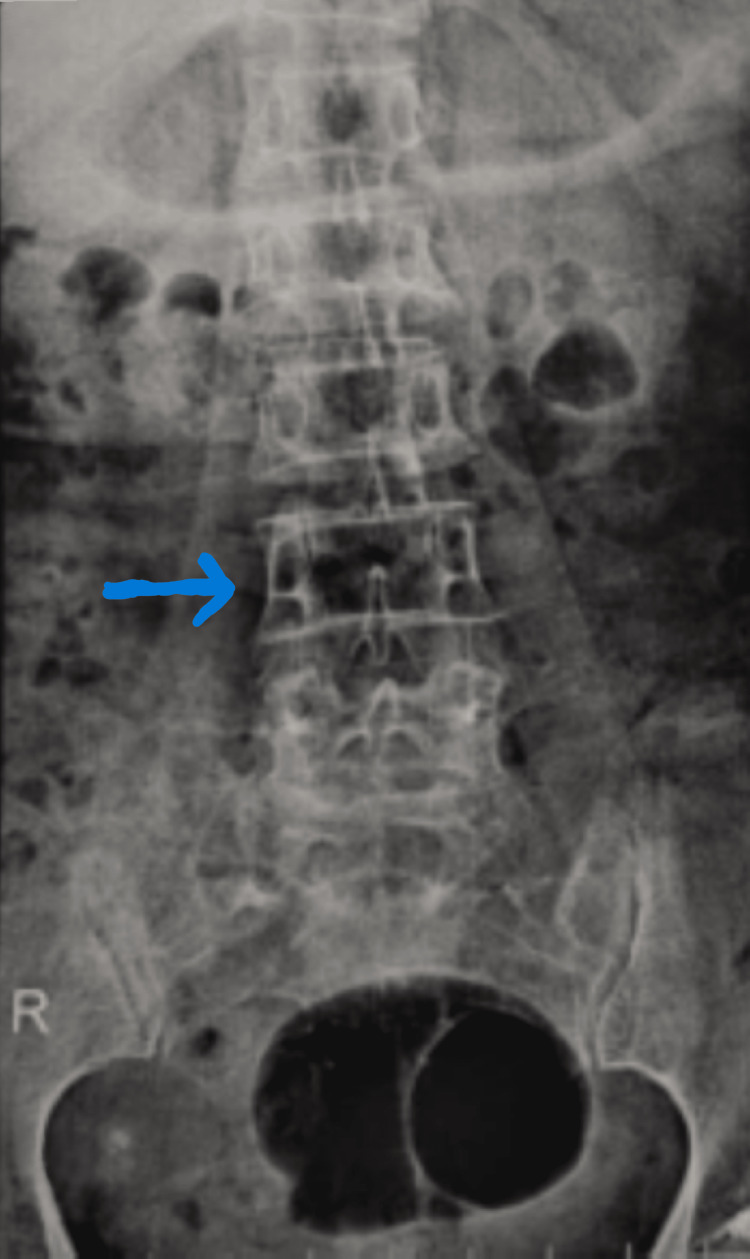
X-ray of the dorso-lumbar spine showing no signs of metastasis (anteroposterior view)

An X-ray of the dorso-lumbar spine imaging showed no lytic lesions in the body of the vertebrae, the image of which has been depicted in Figure 2. USG abdomen was done, which showed a normal liver and no other significant findings. Along with this radiological and metastatic investigation, it was planned for the patient to undergo a modified radical mastectomy, and the same was done.

**Figure 3:**
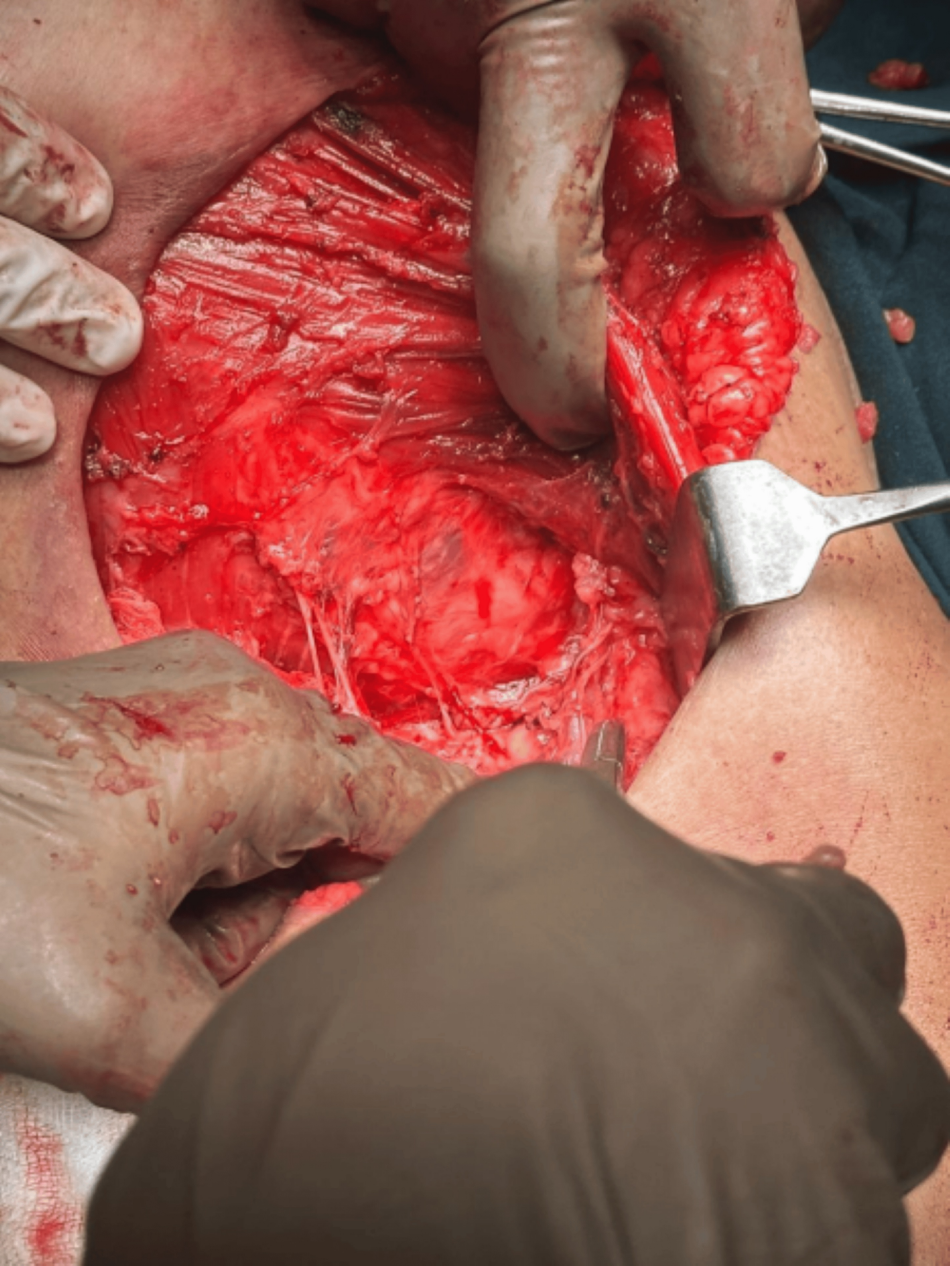
Intraoperative photograph post modified radical mastectomy

Intraoperatively, the entire lump with the breast was removed, and the lump was found to be adherent to the pectoralis muscle, which was excised partly with the lump. The intraoperative picture post resection of the mass has been displayed in Figure 3.

Histopathology of the excised specimen showed that the tumor cells were arranged in the papillary pattern with a central fibrovascular core with a focus on micro invasion at the border. Individual ductal cells showed significant pleomorphism, nuclear hyperchromasia, high nuclear-cytoplasmic ratio, and prominent nucleoli. Immunohistochemistry showed triple negative, solid papillary carcinoma of the left breast. Triple-negative breast cancers are usually an aggressive type of breast cancer with high lymph node positivity. It is associated with a high chance of recurrence.

pTNM classification (American Joint Committee on Cancer (AJCC) 8th edition) was pT2 pNO pM0 (lymphovascular invasion - not identified, perineural invasion - not identified). The patient was initiated on chemotherapy after the wound showed healthy margins after two weeks and is now on regular follow-up. pTNM stands for pathological tumor size, nodal status, and metastatic stage according to the AJCC.

## Discussion

Papillary carcinoma of the breast, although rare, presents unique challenges in diagnosis, treatment, and prognosis. Its distinct histopathology features, coupled with the limited understanding of its molecular biology, contribute to the complexities surrounding this subtype of breast carcinoma.

MRI may show asymmetrical enhancing nodules or complex cysts in papillary carcinomas. IPC morphology and kinetic curves vary on MRI. MRI maps numerous papillary lesions pre-operatively, improving surgical planning [4]. In our case, we directly proceeded to surgery post mammogram and biopsy.

In our case, USG-guided FNAC was done, which showed atypical cells in the sample. A Tru-Cut biopsy was done, which suggested the same features of atypical cells without clear evidence of malignancy. As per various literature, sonography-guided vacuum-assisted core biopsy beats aspiration cytology in proving the features of atypical cells of papillary carcinoma [5].

Immunohistochemistry (IHC) is highly valuable in assessing the absence of myoepithelial cells in invasive cancers. Notably, myoepithelial cells are absent in almost all of the invasive cancers. Several established myoepithelial markers, including S-100, Calponin, CD10, smooth muscle myosin heavy chain, alpha-smooth muscle actin, P63, and high molecular weight cytokeratin, exhibit varying sensitivities and specificities. Among these, P63 and smooth muscle myosin are particularly specific. Myoepithelial markers are predominantly negative in several types of breast malignancy. In our case, these myoepithelial markers were noted to be absent in IHC, representing the aggressive clonal proliferation of tumor cells [6]. IHC for estrogen receptor (ER), progesterone receptor (PR), and human epidermal growth factor receptor 2 (HER2) stood negative in our case [6].

As discussed earlier in the introduction, there exist invasive and non-invasive types of papillary carcinoma, but our case had features of multiple foci of micro invasion in the histopathology of the final specimen, which warranted the modified radical mastectomy that was done in our case [7,8]. Usually, papillary lesions are diagnosed on core biopsy and usually fall in the category of BI-RADS category 3 lesions, necessitating surgical excision for accurate diagnosis [9]. In our case, though the initial FNAC and Tru-Cut biopsy were not suggestive of malignancy, we proceeded with a modified radical mastectomy in view of the mammography and the clinical findings, which were more toward malignancy. Since the number of cases encountered is on the lower side, there have been no large studies conducted exclusively for papillary carcinoma that can decide when breast conservation or modified radical mastectomy has to be done [8]. 

The treatment options are wide local excision, modified radical mastectomy with or without adjuvant radiation, and chemotherapy. Tamoxifen is a significant medication since this cancer is likely hormone-positive and HER2-negative [10,11]. In our case, the patient had a triple negative type of papillary carcinoma, making it a rarity. 

El Sheikh et al. in their study had mentioned that most papillary lesions with no duct invasion and the entire lesion confined to the breast tissue within 1.5 cm distance from the nipple fall under the category of papilloma; if they invade the duct and extend more than 1.5 cm distance from the nipple into the breast parenchyma, then it is more likely to be a papillary malignancy. In our case, the mass filled the duct and extended outside the area of origin into the breast tissue for a distance of more than 1.5 cm from the nipple [12]. Ultrasound and MRI can diagnose breast-invasive papillary carcinoma to a better extent.

Several new treatment modalities have been developed for breast carcinoma of all histological types. While for hormone receptor-positive types, endocrine therapy remains the mainstay treatment, triple negative type of breast cancer requires targeted therapy [13]. Newer drugs such as inhibitors of PARP(poly (ADP-ribose) polymerase), CDK4/6 (cyclin-dependent kinase (CDK)4 and CDK6), PI3K (phosphatidylinositol 3-kinase)/AKT (Ak strain transforming or protein kinase B)/mTOR (mammalian target of rapamycin), multiple kinases, and immune checkpoints have been evaluated and developed for the above [14-16]. However, in papillary carcinoma specifically, surgery and chemotherapy still remain the treatment of choice [17].

Our patient's excised breast specimen with axillary dissection revealed that the tumor cells were arranged in the papillary pattern with a central fibrovascular core and a focus of microinvasion at the border. Individual ductal cells showed significant pleomorphism, nuclear hyperchromasia, high nuclear-cytoplasmic ratio, and prominent nucleoli. Immunohistochemistry showed triple-negative, solid papillary carcinoma of the left breast [18]. The clinicopathological findings of micropapillary carcinoma in another study revealed a high ER and PR positivity ratio as compared to invasive ductal carcinoma, not otherwise specified. In another study, ER positivity ranged from 19.4 to 90.6% in invasive micropapillary carcinomas [19]. One of the studies also indicated that high PR positivity in invasive micropapillary carcinomas is advantageous in terms of survival and attributable to increased life expectancy in these patients [20].

pTNM classification (AJCC 8th edition) in our case was pT2 pNO pM0 (lymphovascular invasion - not identified, perineural invasion - not identified). The patient began chemotherapy after the wound displayed healthy margins two weeks post-operation and is currently undergoing regular follow-up [21].

## Conclusions

There are various histologically high-grade breast malignancies like the metaplastic variant, the medullary type, and papillary carcinoma of the breast. Each has its own way of presentation in terms of hormonal receptors and in classification within the luminal category. Papillary carcinoma, although rare and aggressive, demonstrates a comparatively better prognosis in numerous observational studies compared to medullary or metaplastic types. Additionally, it has been identified as a significant factor in male breast carcinoma, exhibiting heightened aggressiveness compared to its manifestation in females. Enhanced diagnostic methodologies like MRI and IHC (image-guided biopsy) enable early detection, thereby reducing morbidity and mortality rates in both genders.
